# A new highly sensitive enzyme-linked immunosorbent assay for the detection of *Plasmodium falciparum* histidine-rich protein 2 in whole blood

**DOI:** 10.1186/s12936-018-2545-5

**Published:** 2018-11-01

**Authors:** Ihn Kyung Jang, Smita Das, Rebecca S. Barney, Roger B. Peck, Andrew Rashid, Stephane Proux, Emmanuel Arinaitwe, John Rek, Maxwell Murphy, Katherine Bowers, Samuel Boadi, Julie Watson, Francois Nosten, Bryan Greenhouse, Peter L. Chiodini, Gonzalo J. Domingo

**Affiliations:** 1 Diagnostics Program, PATH, Seattle, WA USA; 2 Shoklo Malaria Research Unit, Mahidol-Oxford Tropical Medicine Research Unit, Faculty of Tropical Medicine, Mahidol University, Mae Sot, Thailand; 3 Infectious Disease Research Collaboration, Kampala, Uganda; 4 University of California San Francisco, San Francisco, CA USA; 5 Hospital for Tropical Diseases, London, UK; 6 Centre for Tropical Medicine and Global Health, Nuffield Department of Medicine Research Building, University of Oxford, Old Road Campus, Oxford, UK; 7 London School of Hygiene and Tropical Medicine, London, UK

**Keywords:** *Plasmodium falciparum*, Malaria, Enzyme-linked immunosorbent assay, Histidine-rich protein 2, Elimination

## Abstract

**Background:**

The detection of submicroscopic infections in low prevalence settings has become an increasingly important challenge for malaria elimination strategies. The current field rapid diagnostic tests (RDTs) for *Plasmodium falciparum* malaria are inadequate to detect low-density infections. Therefore, there is a need to develop more sensitive field diagnostic tools. In parallel, a highly sensitive laboratory reference assay will be essential to evaluate new diagnostic tools. Recently, the highly sensitive Alere™ Malaria Ag P.f ELISA (HS ELISA) was developed to detect *P. falciparum* histidine-rich protein 2 (HRP2) in clinical whole blood specimens. In this study, the analytical and clinical performance of the HS ELISA was determined using recombinant *P. falciparum* HRP2, *P. falciparum* native culture parasites, and archived highly pedigreed clinical whole blood specimens from Karen village, Myanmar and Nagongera, Uganda.

**Results:**

The HS ELISA has an analytical sensitivity of less than 25 pg/mL and shows strong specificity for *P. falciparum* HRP2 when tested against *P. falciparum* native culture strains with *pfhrp2* and *pfhrp3* gene deletions. Additionally, the Z′-factor statistic of 0.862 indicates the HS ELISA as an excellent, reproducible assay, and the coefficients of variation for inter- and intra-plate testing, 11.76% and 2.51%, were acceptable. Against clinical whole blood specimens with concordant microscopic and PCR results, the HS ELISA showed 100% (95% CI 96.4–100) diagnostic sensitivity and 97.9% (95% CI 94.8–99.4) diagnostic specificity. For *P. falciparum* positive specimens with HRP2 concentrations below 400 pg/mL, the sensitivity and specificity were 100% (95% CI 88.4–100) and 88.9% (95% CI 70.8–97.6), respectively. The overall sensitivity and specificity for all 352 samples were 100% (CI 95% 96–100%) and 97.3% (CI 95% 94–99%).

**Conclusions:**

The HS ELISA is a robust and reproducible assay. The findings suggest that the HS ELISA may be a useful tool as an affordable reference assay for new ultra-sensitive HRP2-based RDTs.

## Background

Malaria is a vector-borne disease of major public health relevance worldwide. In 2015, the World Health Organization (WHO) reported 212 million new cases and 429,000 deaths, most of which were attributed to *Plasmodium falciparum* [1]. Significant reductions, however, occurred from 2010 to 2015, leading to 21% and 29% decreases in incidence and mortality, respectively [1]. These improvements in disease burden have been largely attributed to vector control, and improved and accessible diagnostics and treatment [1]. As a result, at least 21 countries are positioned for elimination with many others following suit [1, 2], but in order to maintain such progress, continued commitment to malaria control strategies will be required. In particular, the role of current diagnostics has become an increasingly important issue as low density infections have been identified at high rates in low prevalence settings [2, 3]. These low density parasite infections serve as reservoirs and are predicted to make up 20–50% of human-to-mosquito transmission [4]. While RDTs and microscopy are considered the current diagnostic standards for malaria, the limit of detection (LoD) for each tool, 5 parasites/µL in expert reference laboratories and 20 parasites/µL more generally for blood film microscopy; 100–200 parasites/µL and 800 picograms (pg)/mL histidine-rich protein 2 (HRP2) for *P. falciparum* RDTs, is not sufficient for detecting low density infections [4–10]. Highly sensitive and specific field deployable diagnostic tools to detect low density infections may provide more accurate estimates of ongoing malaria transmission as well as render case detection-based elimination strategies more effective. The Alere™ ultrasensitive *P. falciparum* HRP2-based RDT with a greater than tenfold improvement in limit-of-detection for HRP2 over previously available RDTs was launched in April 2017 [11, 12]. The development and performance of these tools for low density infections also require complementary laboratory-based reference assays for the same analytes that can confirm performance of these tests.

Currently available standard ELISAs for HRP2 do not attain low enough LoDs for HRP2 to serve as useful reference assays for new highly sensitive HRP2-based RDTs or to define HRP2 distributions in populations with a large proportion of low density infections [13, 14]. While suitably sensitive assays for HRP2 already exist, the platforms are not readily available to laboratories outside the research context [11, 15]. A standard ELISA remains the most accessible platform to most laboratories. A novel highly sensitive Alere™ Malaria Ag *Pf* ELISA (HS ELISA) has been developed. The HS ELISA has a similar platform and protocol compared to current commercial *P. falciparum* HRP2-based ELISA kits, but requires a smaller volume of blood, 50 μL versus 100 μL, respectively, making it an attractive reference tool for large-scale use in field laboratories. In this study, HS ELISA performance against *P. falciparum* HRP2 was characterized using *P. falciparum* recombinant HRP2, a panel of *P. falciparum* native culture specimens, and clinical whole blood specimens from Myanmar and Uganda.

## Methods

### Human subjects research

All study participants provided consent for whole blood specimen collection as part of studies approved by institutional review boards (IRBs). Specimens from Karen Village (TOT), Myanmar were collected and approved by OxTREC (Reference No. 1017-13 and 1015-13), by Tak Community Advisory Board, and local village committees. Ugandan specimens from Nagongera were collected under a study approved by the University of California San Francisco (UCSF) (IRB No. 11-05995), Makerere University (IRB No. 2011-0167), and London School of Hygiene and Tropical Medicine (LSHTM) (IRB No. 5943). Prior to PATH (Seattle, Washington, USA) receiving the specimens, all specimens were delinked and anonymized for analysis as described in consent forms.

### Highly sensitive *Plasmodium falciparum* ELISA (HS ELISA)

The presence or absence of *P. falciparum* HRP2 was determined by the Alere™ Malaria Ag P.f (HRP2) ELISA (HS ELISA) (Reference Number: 05EK10; Republic of Korea) according to the manufacturer’s guidelines. The HS ELISA kit includes a capture antibody-coated 96-well plate, sample diluent, conjugate diluent, 1st enzyme conjugate concentrate, 2nd enzyme conjugate concentrate, a cutoff calibrator, positive and negative controls, Tetramethylbenzidine (TMB) substrate, stop solution, washing buffer concentrate and adhesive plate film. Briefly, 50 µL of blood, calibrator and controls were added to capture antibody-coated wells in duplicate, that contain 50 µL of sample diluent and then incubated at 37 °C for 60 min. The wells were washed to remove unbound blood proteins with washing buffer. The diluted 1st enzyme conjugate (100 μL) was then added and incubated for 1 h. After washing, incubation with the diluted 2nd enzyme conjugate was followed. A solution of TMB substrate was added after a final wash to remove unbound conjugate and the resultant color change was measured by SpectraMax i3x (Molecular Devices, Sunnyvale, California, USA). After subtracting absorbance value at 620 nanometer (nm) from absorbance value at 450 nm, replicate signal values were averaged and the assay results were interpreted as positive or negative relative to the cutoff absorbance. The HS ELISA results were considered “positive” if the average absorbance value was equal or above the calculated cutoff, “negative” if the average absorbance was below the calculated cutoff, or “discordant” if a single replicate absorbance value was above the cutoff (“positive”) whereas the other replicate absorbance value was below the cutoff (“negative”) with a coefficient of variation (CV) above 15%. Discordant results were retested on a new plate if adequate sample volumes were available.

### Analytical sensitivity and specificity

To determine the analytical sensitivity of HRP2 by the HS ELISA, *P. falciparum* recombinant GST-W2 HRP2 (rGST-W2) (Microcoat Biotechnologie GmbH, Bernried am Starnberger See, Germany; Catalog # 30081) was obtained and serially diluted twofold in negative whole blood (BioreclamationIVT, Hicksville, New York, USA) from 800 to 5 pg/mL HRP2. At first, the limit of blank (LoB) and limit of detection (LoD) were determined with results from ten assays according to the Clinical and Laboratory Standard Institute (CLSI) EP-17A guidance: LoB = mean_blank_ + 1.645 (SD_blank_), LoD = LoB + 1.645 (SD_low concentration sample_) [16]. LoB and LoD were determined to be 0.11 and 0.13 by absorbance, respectively. The cutoff calibrator was formulated on the basis of the calculated LoD in absorbance. Performance of the calibrator was then assessed by testing dilution samples in duplicate with a cutoff calibrator for thirteen ELISA runs and determining positive and negative for HRP2.

For analytical specificity, the following seven *P. falciparum* native culture strains were used for testing: ITG, W2, 3D7, Dd2, D10, HB3 from BEI Resources (Manassas, Virginia, USA) and 3BD5 from National Institute of Allergy and Infectious Diseases (NIAID, Bethesda, Maryland, USA). The wild-type strains, ITG, W2, 3D7, contain both the *pfhrp2* and *pfhrp3* genes, whereas Dd2 and D10 *are pfhrp2*−*/pfhrp3*+ and HB3 is *pfhrp2*+*/pfhrp3*−. The 3BD5 strain lacks both *pfhrp2* and *pfhrp3* genes (double deletion). All culture strains were cultured in vitro at the PATH laboratory [17]. Synchronization of cultures was performed by sorbitol-treatment procedure [18]. Parasitaemia was measured by light microscopy with a 100× oil objective by two trained microscopists. All *P. falciparum* native culture strains were serially diluted twofold in negative whole blood (BioreclamationIVT, Hicksville, New York, USA) from 2000 to 0.01 p/µL and tested in duplicate by the HS ELISA. All specimens were stored at − 80 °C and thawed on ice during testing. For each dilution, a normalized absorbance ratio (NAR) was measured by dividing the calculated absorbance value with the average absorbance of the calibrator on the sample plate.

### Reproducibility and variability

The reproducibility of the HS ELISA were determined by the Z′-factor statistical method [19]. The Z′-factor method measures suitability of an assay for high-throughput screening by measuring and comparing the means and standard deviations of positive and negative controls. *P. falciparum* 3D7 (*pfhrp2*+, *pfhrp3*+*)* and *P. falciparum* 3BD5 (*pfhrp2*−*, pfhrp3*−) culture strains were used as the positive and negative controls respectively. The Z′-factor experiment was performed with the positive (3D7) and negative (3BD5) controls diluted in negative whole blood and consisted of 20 positive and 20 negative replicates tested on each day for 2 days. Calculated Z′-values between 0.5 and 1 indicated an excellent assay and values between 0 and 0.5 were considered as a marginal assay. The formula for the Z′-factor calculation is the following:

$$ \begin{aligned}Z^{\prime}{\text{-factor}} &= 1 - \left( {3*{\text{SD}}\;\text{positive}\;\text{control + }3*{\text{SD}}\;\text{negative}\;\text{control}} \right) \\ & \quad /  \left( {{\text{mean}}\;{\text{positive}}\;{\text{control}} - {\text{mean}}\;{\text{negative}}\;{\text{control}}} \right) \end{aligned}$$

SD: standard deviation.

For assay variability, negative whole blood was prepared with 3D7 infected red blood cells (RBCs) to a single stock concentration of 8.23 p/µL. The stock specimen was stored at − 80 °C and thawed on ice before testing. The specimen was tested on each of 5 days, with ten replicates per plate. The % CVs were calculated to describe intra- and inter-plate variability. A mean % CV at or below 15% for interplate variability and a mean % CV at or below 10% for intraplate variability suggested excellent consistency in assay results.

### Diagnostic sensitivity and specificity

Clinical whole blood specimens were collected from asymptomatic study participants in Myanmar and Uganda, as previously described [11]. Both study sites were selected because they represent different malaria endemic settings; the asymptomatic *P. falciparum* infection rate was 1.9% in Myanmar (May–April 2015) and 43% in Uganda (May–September 2015) [11]. In Myanmar, both children and adults were recruited during two consecutive routine household visits as part of a study to assess mass drug administration (MDA) for malaria elimination [11]. In Uganda, children 6 months to 11 years and their primary givers were recruited across 100 random households as part of an ongoing cohort study [11]. The study participants visited a health clinic every 3 months for routine visits, at which venipuncture blood collection and microscopy were performed. Participants were recruited based on asymptomatic status, which was defined as body temperature less than 37.5 °C, no malaria treatment within the previous 60 days, and no other clinical symptoms of malaria [11]. Venipuncture whole blood (1.5–2 mL in EDTA vacutainer tubes) was collected from each participant [11]. The blood was aliquoted and frozen at − 80 °C in the field and transported frozen to PATH (Seattle, Washington, USA), where the samples were stored at temperatures − 80 °C until testing [11].

Diagnostic performance using clinical specimens was determined in two parts; first, *P. falciparum* positive and negative clinical specimens from Myanmar and Uganda were chosen based on *P. falciparum* microscopy and confirmed by qRT-PCR [11]. Second, *P. falciparum* positive clinical specimens from Uganda were selected based on qRT-PCR and previously characterized HRP2 concentrations below 400 pg/mL [11], the LOD of the current commercial Malaria Ag CELISA (Cellabs Pty. Ltd, Brookvale, New South Wales, Australia).

*Plasmodium falciparum* negative specimens were also from Uganda and chosen based on microscopy and confirmed by qRT-PCR. For part one, 300 specimens were selected, of which 100 positive specimens were from Uganda and 200 negative specimens from Myanmar. For part two, 60 specimens were tested, of which 30 specimens were positive and 30 specimens were negative. Before HS ELISA testing, 120 µL of each specimen was taken from the master aliquot, randomized, and blinded to hide the identity of the sample from the test operator. These archived frozen whole blood specimens experienced no more than three freeze–thaw cycles before testing. Specimens were defrosted immediately prior to use and were stored on ice during testing. Each specimen was tested in duplicate. Specimen identification was subsequently unblinded to match test results to the reference method and determine HS ELISA diagnostic performance.

### Statistical analysis

GraphPad Prism, version 6.0 (GraphPad, California, USA) was used for statistical analysis of HS ELISA absorbance values, analytical sensitivity and specificity, and reproducibility and variability studies. For diagnostic sensitivity and specificity using clinical specimens from Myanmar and Uganda, the sensitivity was calculated by (true positives)/(true positives + false negatives) and the specificity by (true negatives)/(true negatives + false positives). The 95% confidence intervals were measured by exact binomial method in Stata 14.2 (College Station, Texas, USA).

## Results

### Analytical sensitivity and specificity

**Fig. 1:**
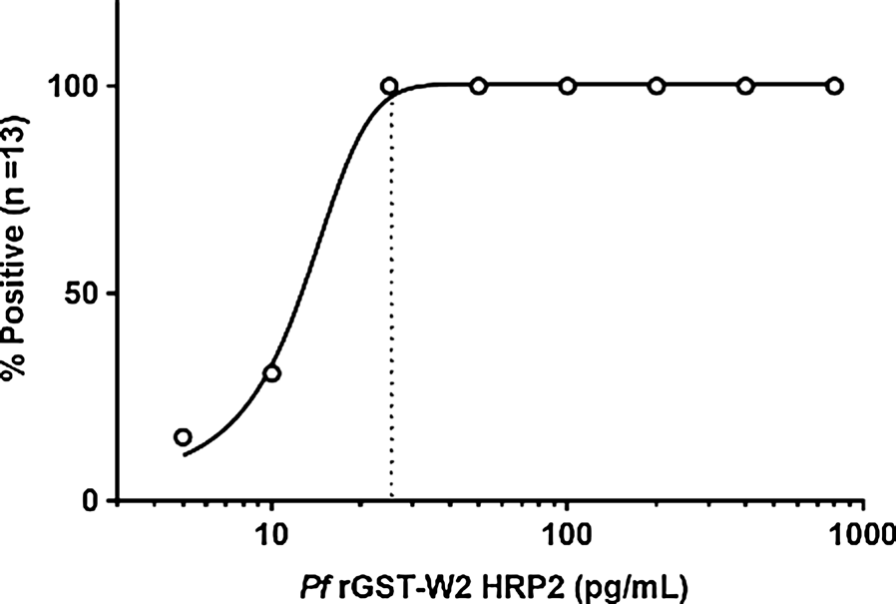
Analytical sensitivity of the *Plasmodium falciparum* HS ELISA for the detection of HRP2 in whole blood. Recombinant *P. falciparum* rGST-W2 HRP2 specimens at concentrations ranging from 5 to 800 pg/mL were tested by HS ELISA in duplicate per run and over 13 runs. The absorbances of each replicate per concentration were averaged and interpreted as positive or negative relative to the cutoff absorbance for each plate. The results were plotted based on the percentage of positive tests associated with each target concentration

A series of rGST-W2 HRP2 specimens ranging from 5 to 800 pg/mL HRP2 and negative whole blood controls were tested in duplicate for 13 ELISA runs to characterize HS ELISA sensitivity. The results demonstrated that the HS ELISA was able reliably to detect 100% of whole blood specimens with 25 pg/mL HRP2 (Fig. 1). Four out of the 13 samples (30.8%) were positive at 10 pg/mL, and 2 out of 13 samples (15.4%) were positive at 5 pg/mL. All negative controls lacked reactivity.

The analytical specificity of the HS ELISA was investigated using seven *P. falciparum* native culture strains (ITG, W2, 3D7, Dd2, D10, HB3 and 3BD5) and the results were analysed for normalized absorbance ratios at different parasite concentrations (Table 1). At 74.1 p/µL, the wild-type strains (3D7, ITG, W2) and *hrp3* deletion strain (HB3) showed high ratios (31.8–40.1), whereas the *hrp2* deletion strains (Dd2, D10) showed lower ratios (1.5–2.9) (Table 1). The parasite strain 3D7 was detected as low as 0.01 p/µL. Assay reactivity to the *hrp2/hrp3* double deletion strain (3BD5) was not observed in any of the parasite concentrations (Table 1). The HS ELISA was also capable of detecting wild type strains and the *hrp3* deletion strain as low as 0.01–0.9 parasites/µL and 0.1 parasites/µL, respectively (Table 1).

**Table 1:** Normalized absorbance ratios of seven *Plasmodium falciparum* native culture strains (ITG, W2, 3D7, HB3, Dd2, D10, 3BD5) for parasitaemia ranging from 0.01 to 2000 p/µL

Parasitaemia (p/µL)	Normalized absorbance ratio						
	ITG	W2	3D7	HB3	Dd2	D10	3BD5
2000	43.9	44.1	40.1	31.8	40.1	31.8	0.8
666.7	44.1	44.1	40.1	31.8	32.2	23.5	0.8
222.2	44.0	44.0	40.1	31.8	10.0	7.3	0.8
74.1	32.1	37.3	40.1	31.8	2.9	1.5	0.8
24.7	16.0	19.2	40.2	28.9	1.0	0.7	0.8
8.2	6.2	8.0	31.3	13.7	0.8	0.6	0.8
2.7	2.5	3.4	17.0	6.0	0.9		0.9
0.9	1.5	1.9	7.5	3.0	0.8		0.8
0.3	0.9	1.2	3.5	1.6			
0.1	0.8	0.9	2.3	1.0			
0.03	0.8	0.9	1.5	0.8			
0.01	0.7	0.9	1.3	0.5			

The ITG, W2, and 3D7 strains have both *hrp2* and *hrp3* genes, HB3 is an *hrp3* deletion strain, Dd2 and D10 are *hrp2* deletion strains, and 3BD5 is an *hrp2* and *hrp3* double strain

### Reproducibility and variability

Two independent experiments were performed using the Z′-factor statistical method to evaluate HS ELISA reproducibility. Using the mean and standard deviation values for *P. falciparum* 3D7 and *P. falciparum* 3BD5 positive and negative replicate wells, the positive control % CV was 1.37% [standard deviation (SD): 0.02] and the negative control % CV was 9.72% (SD: 0.01). The average Z′-factor was 0.862. The inter-plate variability using *Pf* 3D7 strain showed a mean % CV of 11.76%. The intra-plate variability, also using *Pf* 3D7, had a % CV of 2.51%.

### Diagnostic sensitivity and specificity

In part one, a total of 300 clinical whole blood specimens from malaria endemic areas were selected for evaluating HS ELISA diagnostic performance. Two-hundred *P. falciparum* negative specimens from Myanmar and 100 *P. falciparum* positive specimens from Uganda were chosen based on *P. falciparum* microscopy and qRT-PCR results. The parasitaemia in the positive specimens ranged from 8.84 to 235,095.8 p/µL. The mean and median parasitaemia were 8205 p/µL and 1090 p/µL, respectively. The *P. falciparum* HRP2 concentrations in the positive specimens ranged from 25.2 to 14,600 pg/mL (14,600 pg/mL was the upper LOD reported previously [11]). The mean and median HRP2 concentrations were 9999 pg/mL and 10,851.3 pg/mL respectively. From the initial testing of the clinical specimens using the HS ELISA, 104 specimens were positive, 173 specimens were negative, and 23 specimens were discordant. Of the discordant specimens that met the criteria for retesting, five lacked the volume in the master aliquot for retesting and were subsequently removed from the final sensitivity and specificity calculations. The remaining 18 specimens tested as negative after repeat testing. The HS ELISA sensitivity and specificity were 100% (CI 95% 96.4–100%) and 97.9% (CI 95% 94.8–99.4%) respectively (see Table 2).

**Table 2:** HS ELISA performance using *Plasmodium falciparum* positive and negative clinical specimens from Uganda and Myanmar that were (A) confirmed by composite microscopy and qRT-PCR and (B) confirmed by qRT-PCR and HRP2 concentrations below 400 pg/mL

Panel A					
		Microscopy and qRT-PCR		Sensitivity (95% CI)	Specificity (95% CI)
		Positive	Negative		
HS ELISA	Positive	100	4	100% (96.4–100%)	97.9% (94.8–99.4%)
	Negative	0	191		

Panel B					
		qRT-PCR and HRP2 < 400 pg/mL		Sensitivity (95% CI)	Specificity (95% CI)
		Positive	Negative		
HS ELISA	Positive	30	3	100% (88.4–100%)	88.9% (70.8–97.6%)
	Negative	0	24		

In part two, a total of sixty clinical whole blood specimens from Uganda were tested for diagnostic performance using the HS ELISA. Thirty specimens were *P. falciparum* positive by qRT-PCR and also had HRP2 concentrations less than 400 pg/mL, and an additional 30 specimens were *P. falciparum* negative based on concordant microscopy and qRT-PCR. The *P. falciparum* positive specimens had parasitaemia ranging from 0.04 to 1158.8 p/µL. The mean and median parasitaemia were 90.4 p/µL and 13.1 p/µL, respectively. The HRP2 concentrations in positive specimens ranged from 6.1 to 337.2 pg/mL. The mean and median HRP2 concentrations were 107.4 pg/mL and 73.2 pg/mL, respectively. Of the 60 specimens tested initially using the HS ELISA, three specimens were discordant and had insufficient volume for retesting; these specimens were excluded from performance measurements. The HS ELISA sensitivity and specificity were 100% (CI 95% 88.4–100%) and 88.9% (CI 95% 70.8–97.6%), respectively (see Table 2).

## Discussion

The RDTs and conventional ELISA-based assays are considered to be common methods for malaria detection in both field and laboratory settings, but are both restricted to detecting high HRP2 concentrations. While this has critical importance for identifying and treating acute *P. falciparum* malaria infections, the current challenge of detecting asymptomatic low density infections with correspondingly low HRP2 levels has made these tools inadequate [2, 4, 20]. This is the first report of an analytical and diagnostic validation of a new highly sensitive HRP2-based screening immunoassay against recombinant and native *P. falciparum* HRP2 strains and archived clinical whole blood specimens. Using multiple different specimen types provided a detailed characterization of HS ELISA performance, reproducibility, and variability.

The HS ELISA was shown to be a sensitive and robust assay. The HS ELISA was able to detect 100% of *P. falciparum* rGST-W2 HRP2 at 25 pg/mL, a 16-times improvement over the commercial CELISA (LOD: 400 pg/mL rGST-W2 HRP2). While the HS ELISA was evaluated with rGST-W2 HRP2 an international reference standard for malaria diagnostic evaluations the analytical sensitivity may vary across other recombinant proteins as shown previously with the CELISA and other HRP2 assays [13–15, 21].

The HS ELISA analytical sensitivity results also indicated high assay reactivity to *P. falciparum* HRP2 across a wide range of low parasitaemias, 0.01–74.1 p/µL, as well as a panel of *P. falciparum* native culture strains with different *pfhrp2* and *pfhrp3* genotypes. The assay reactivity was the poorest with *pfhrp2*−*/pfhrp3*+ deletion strains (Dd2, D10) compared to wild-type (ITG, W2, 3D7) and *pfhrp2*+/*pfhrp3*− deletion strain (HB3). *P. falciparum* 3BD5 strain, lacking both *pfhrp2* and *pfhrp3*, was negative by HS ELISA. These findings correlate well with *pfhrp2* and *pfhrp3* genetic detection profiles, assuming some cross-reactivity of the assay with HRP3 as demonstrated for other HRP2 tests. The reproducibility Z′-factor estimate (0.862) was within the range 0–0.5, indicating the HS ELISA as a robust and reproducible assay. The inter-assay and intra-assay variability were within the acceptable range of % CVs, less than 15% and less than 10%, respectively. Together, these results suggest the HS ELISA to be a robust and reproducible assay. Additional studies are required to further expand the Z′-factor estimate, inter-assay and intra-assay variability studies to other *P. falciparum* strains and in different locations. Parallel studies comparing the performances of conventional ELISAs and the new HS ELISA with *P. falciparum* recombinant and native culture strains would also further define the capacity of the HS ELISA to discriminate positive and negative results.

An ELISA that is able to detect low levels of *P. falciparum* HRP2 is a valuable reference assay to detect and confirm asymptomatic low density infections. These infections not only have low parasite densities, but also low HRP2 levels, and are thus difficult to identify with current field methods, RDT and microscopy [5–7, 22]. The recognition that more sensitive field tools are vital for continued success of malaria programmes has encouraged the development of next-generation diagnostic tests [2, 11]. The HS ELISA may serve as a reference tool to confirm the results of these novel diagnostics. Here, HS ELISA performance was characterized using clinical whole blood specimens from asymptomatic participants in Myanmar and Uganda, representing low and high transmission areas, respectively. Using these specimens, the HS ELISA sensitivity and specificity in Part 1, 100% and 97.9% respectively, and in Part 2, 100% and 88.9% respectively, were excellent. It should be noted especially for Part 2 because of the small sample size (n = 60) that three discordant specimens were excluded from analysis because of insufficient volume for re-testing, which could have biased the sensitivity or specificity measurements. Additionally, the clinical specimens were not tested for *hrp2* and *hrp3* deletions, which if present, would have likely influenced performance results. Future studies should recognize this limitation of the HS ELISA and test for *hrp2* and *hrp3* deletion parasites concurrently.

The utility of the HS ELISA could potentially extend beyond a reference test for malaria control and elimination programmes; the assay could be used also in drug sensitivity trials, possibly in blood bank screening, though a negative would not guarantee complete absence of parasites, and other epidemiological studies [14, 23, 24]. The performance and impact of the HS ELISA for these other applications should be investigated.

## Conclusions

The HS ELISA demonstrated acceptable sensitivity and specificity for detecting *P. falciparum* HRP2, including recombinant protein, native culture *P. falciparum* parasites, and clinical whole blood specimens. This new assay will be useful in assessing new diagnostic tools and possibly other malaria intervention trials due to its lower limit of detection of *P. falciparum* HRP2. With an increased number of reports of *pfhrp2/pfhrp3* gene deletions resulting in false negative results, future work should focus on the development of a more sensitive ELISA to identify parasites with *pfhrp2/pfhrp3* deletions and possibly other *Plasmodium* malaria species.

## Abbreviations

CELISA: Cellabs Malaria Ag ELISA
CV: coefficient of variation
ELISA: enzyme-linked immunosorbent assay
HRP2: histidine-rich protein 2
HS ELISA: highly sensitive Alere™ Malaria Ag P.f ELISA
LoB: limit of blank
LoD: limit of detection
NAR: normalized absorbance ratio
p/µL: parasites per microlitre
pg/mL: picogram per millilitre
*pfhrp2*: *Plasmodium falciparum* histidine-rich protein 2 gene
*pfhrp3*: *Plasmodium falciparum* histidine-rich protein 3 gene
RDT: rapid diagnostic test
SD: standard deviation
rGST-W2: *Plasmodium falciparum* recombinant GST-tagged W2
